# Using posterior probability informed thresholds to develop best practice recommendations for MorphoPASSE using the innominate, cranial, and combined traits

**DOI:** 10.1111/1556-4029.70192

**Published:** 2025-10-12

**Authors:** Kate M. Lesciotto, Alexandra R. Klales

**Affiliations:** ^1^ University of North Texas Health Science Center Fort Worth Texas USA; ^2^ Washburn University Topeka Kansas USA

**Keywords:** best practice recommendations, cranial traits, forensic anthropology, innominate traits, MorphoPASSE, posterior probability informed thresholds, sex estimation

## Abstract

MorphoPASSE is a free program that estimates sex based on morphological traits of the innominate, cranium, or a combined set of traits; however, MorphoPASSE does not provide recommendations on which set of traits performs best or recommend a posterior probability (PP) threshold for sex classification in modern forensic casework. The goals of this study were to compare accuracy rates when using different sets of traits and when imposing posterior probability informed thresholds (PPITs). Innominate and cranial trait score data were collected from four modern US documented skeletal collections (*n* = 285). Accuracy rates for five mutually exclusive PP intervals were calculated, and PP intervals that were significantly different from chance were condensed into PPITs. Using a PPIT of 0.85–1.00 produced high accuracy rates of 97.3% for the innominate traits and 90.2% for the cranial traits. Using the combined set of innominate and cranial traits resulted in significantly higher accuracy (99.6%) with a lower PPIT of 0.75–1.00. Additionally, the combined trait model corrected all previous misclassifications by either producing a correct sex classification or leaving the individual unclassified for failing to reach the required PPIT. Therefore, when both elements are available, the combined set of traits is recommended with a 0.75–1.00 PPIT. Individuals with a PP falling between 0.75 and 1.00 should be reported as consistent with the male or female reference samples, while those falling below should be reported as “could not be estimated.” Use of these recommendations will help standardize the use of MorphoPASSE and reporting sex estimation results.

**Highlights**

- Using the combined set of innominate and cranial traits in MorphoPASSE reduced sex bias.
- PPITs resulted in higher accuracy rates when using all three sets of traits.
- The recommended PPITs reduced the potential for misclassifying an individual with high confidence.

## 1 INTRODUCTION

Estimation of the biological profile—an individual's age‐at‐death, sex, ancestry or population affinity, and stature—is a core component of forensic anthropology practice. Even with advances in DNA analysis, methods for sex estimation from skeletal remains continue to play a crucial role in early investigations, particularly to help narrow down the potential decedent pool. Although both sex and gender are not binary and exist along a spectrum, forensic anthropological methods for sex estimation are based on a binary classification scheme. Forensic anthropology practitioners use a wide variety of sex estimation methods, including methods that utilize osteometric and morphological data. When using morphological traits for sex estimation, the innominate and skull are considered the most informative skeletal elements [1].

The most frequently used skull traits are glabella (G), nuchal crest (NC), mastoid process (MP), supraorbital margin (SOM), and mental eminence (ME). Buikstra & Ubelaker [2] recommended scoring these traits on an ordinal scale of 1–5, although sex estimation was based on an overall assessment. Walker [3] was the first to integrate these traits into a statistical framework, providing six discriminant functions using either two or three of the traits, based on pooled data from African American, European American, and English individuals. The accuracy rates of these equations ranged from 76.8–88.4%. Along with the discriminant functions, Walker [3] also provided formulae for calculating the posterior probability (PP) of the individual being male or female.

While an improvement on the overall assessment approach with these traits, the Walker [3] method is not without issues. The six discriminant functions include MP or ME in every equation. Therefore, the method could not be used for individuals with damage or pathology to MP and ME. Additionally, Walker [3] only provided text descriptions of the extreme presentations of each trait (scores 1 and 5) and basic diagrammatic representations of the trait scores. Numerous studies have tested the validity and reliability of the method with mixed results (see, e.g., [4, 5, 6, 7, 8, 9]).

The most frequently used innominate traits [ventral arc (VA), subpubic contour (SPC), and medial aspect of the ischiopubic ramus (MA)] were initially described by Phenice [10] and expanded upon by Klales et al. [11]. Phenice [10] used a presence/absence approach delineated by sex and used a majority rule for sex estimation. Klales et al. [11] presented the same three traits, now ordinally scored on a scale of 1–5, with text descriptions, diagrams, and photographic exemplars of real bone for each trait score. These scores could be used in a logistic ordinal regression equation, and like Walker [3], Klales et al. [11] provided a formula for calculating the PP of sex membership. Also like the Walker [3] method, the Klales et al. [11] method for the innominate was not without limitations. The primary drawback is the inability to use the method if any one of the traits cannot be scored or is damaged.

### 1.1 MorphoPASSE

Recently, the MorphoPASSE [12, 13] program graphical user interface (GUI) has risen in popularity among practicing forensic anthropologists for estimating sex based on these same morphological traits of the skull and innominate [1]. MorphoPASSE revised and expanded the Walker [3] method to include intermediate score descriptions for each trait, added photographs of real bone examples for each trait score, and allowed sex to be estimated using any combination of available traits. Although included in the GUI, MorphoPASSE [12] currently cautions against the use of ME scores due to high interobserver error documented in numerous studies (see, e.g., [5, 6, 7]). The other four traits (G, NC, SOM, and MP) are collectively referred to as the “cranial traits.” MorphoPASSE provided similar images and trait descriptions for the three innominate traits. Among the trait scoring revisions, MorphoPASSE changed the orientation for scoring the SPC to a ventral, rather than dorsal, view and provided considerations for feature weighting within traits (e.g., bone shape in the VA and presence of the ridge or plateau for MA).

In addition to these changes, MorphoPASSE provided several other benefits. First, while still allowing the use of the original Walker [3] and Klales et al. [11] equations, MorphoPASSE offers a random forest model (RFM) option. RFM is a flexible machine learning algorithm that offers the benefits of only selecting the most informative input variables, accommodating missing data, and not making assumptions about the data (e.g., normal distribution, sample size). RFM is the recommended analytical option for MorphoPASSE [13, 14]. Unless otherwise specified, use of the RFM can be assumed for all subsequent discussions about MorphoPASSE and associated outputs.

Second, while the majority of sex estimation methods have focused on a single skeletal region, MorphoPASSE allows users to obtain a sex estimation based on only the cranial traits, only the innominate traits, or a combined set of cranial and innominate traits. The option to include both cranial and innominate traits offers several potential advantages. While it is generally accepted that the innominate is the most sexually dimorphic element [15, 16], the pubis is frequently damaged by taphonomic factors, which could affect the ability to properly score any or all of the innominate traits [17]. Further, a review of 360 cases with sex confirmed through DNA found that sex estimation accuracy improved when more skeletal elements were available for analysis [18]. Lastly, Best et al. [19] determined that there was no significant intraindividual correlation between the innominate and cranium in terms of size, shape, or degree of sexual expression, suggesting that an individual could have a relatively gracile cranium but a relatively robust innominate and vice versa. Currently, MorphoPASSE is the only method that combines data from cranial and postcranial elements into a single sex estimate. However, the MorphoPASSE manual [13] does not provide recommendations on which traits to utilize for classification, aside from avoiding the ME, and it is up to the practitioner to determine whether to test the innominate and cranium separately or use all traits in combination.

For each case analysis, the MorphoPASSE output provides a ‘case prediction’ with the PP of the individual being male (PPM) and the PP of the individual being female (PPF). Since MorphoPASSE is limited to a binary sex classification scheme of male and female, PPM and PPF will always equal a total of 1.0. Classification is based on the greater of the two PP values (i.e., whether PPM or PPF is >0.50). Presumably, an individual with a PPM of 0.99 would be classified in a forensic report as male with more confidence than an individual with a PPM of 0.51; however, the MorphoPASSE manual [13] does not provide a recommended PP threshold for classifying an individual in the context of modern forensic casework. The MorphoPASSE manual provides an example of an output file and simply notes that the *Case Prediction* section “provides the probability of sex membership” [13]. Additional guidance was more recently provided by Klales [14], which, when describing a hypothetical MorphoPASSE output, stated that “a probability of 60% (i.e., close to random chance) should be interpreted as far less meaningful than a probability of membership of 85% or above for sex.” However, the ultimate decision of what PP threshold to set for classification and how the threshold and results should be translated into a case report remains a highly individual decision.

Peer‐reviewed tests of MorphoPASSE are surprisingly limited given its popularity, possibly due to the extensive testing of previous methods using the cranial and innominate traits for sex estimation or because the method is inherently self‐validating. However, revisions of the trait descriptions, use of a different reference sample, and implementation of the RFM require new testing of MorphoPASSE. Kotěrová et al. [20] is the only peer‐reviewed publication that validates MorphoPASSE, reporting an overall accuracy of 85.6%–88.3%, depending on observer, using the four cranial traits with a PP threshold of 0.50 for classification. Multiple published abstracts have reported MorphoPASSE accuracy rates, although studies are difficult to compare due to the lack of published details and range of PP thresholds. MorphoPASSE accuracy rates for sex estimation using the four cranial traits have been reported as 85.4% with a PP threshold of 0.50 [21] and 77.9% with no stated PP threshold [22], although the latter study [22] collected the data using the trait descriptions from Walker [3] rather than the modified descriptions in the MorphoPASSE manual [13]. Accuracy rates when using the three innominate traits have been significantly higher at 93% with no stated PP threshold [23] and 90.2% with a 0.95 PP threshold [24]. The only reported accuracy rate when using the combined set of innominate and cranial traits exceeded 98% when applying an “80% classification threshold” [25], which was interpreted as a PP threshold of 0.80 for classification. Differences in samples, observers, and PP thresholds for classification between these studies make it impossible to compare accuracy rates in MorphoPASSE between models using the cranial, innominate, or combined traits, and no single study has directly compared accuracy rates between the different model options.

### 1.2 Posterior probability thresholds

The issue of appropriate PP thresholds for classification has been a topic of interest in recent years with respect to methods for sex estimation. While most forensic anthropologists are familiar with accuracy rates, multiple recent publications have pointed out the potential confusion between an accuracy rate and the degree of confidence in a sex estimation for a specific individual or set of remains. A method with a 95% accuracy rate does not guarantee that each individual has a PP of 0.95 (i.e., each individual classified by the method has only a 5% chance of being misclassified) [17, 20, 26]. Method accuracy and individual classification PPs are distinct but equally important considerations. The overall accuracy rate influences the decision of which method to select [1, 27], while the PP reflects the confidence in a specific case classification and guides how a practitioner chooses to report the method results in a case report.

There have been several recent attempts to standardize the use of PPs for sex estimation methods and move away from making a classification based simply on whether the male or female PP exceeded random chance of 0.50, regardless of magnitude. Jerkovic et al. [26] argued that rather than applying a standard PP threshold for classification, individual methods should be evaluated to determine a PP threshold that results in a predefined accuracy level, set at 95% for their study. However, their rationale, that a 95% accuracy rate is “required by modern forensic science standards,” is arguably misguided and certainly incorrect for US forensic casework. In the US, *Daubert* sets the standard for the admissibility of expert witness evidence in federal and most state courts. However, many forensic anthropology publications have over‐interpreted *Daubert* as requiring a specific minimum accuracy rate for admissibility, and this simply has no foundation in the actual language of the *Daubert* decision or subsequent decisions related to forensic anthropological expert witness evidence [28].

Alternatively, a method can automatically set a PP threshold, such that the program or GUI will indicate that sex cannot be estimated for any individual that does not meet the required threshold. *Diagnose Sexuelle Probabiliste 2* (“DSP2”) [29] is a sex estimation method that uses between four and ten measurements of innominate and takes this approach, with a set PP threshold of 0.95. While DSP2 has been demonstrated to have high classification accuracies, the high PP threshold significantly reduces the number of individuals that can be classified by the method and also results in a sex bias, with significantly more males reaching the 0.95 threshold and being classified than females [30].

Rather than imposing an automatic, and arguably somewhat arbitrary, PP threshold, Avent et al. [31] suggest using PP *informed* thresholds (PPITs). By studying different threshold levels, Avent et al. [31] investigated how applying PPITs impacts accuracy rates and can provide a more nuanced way to balance the need for high accuracy against the need to maintain a high inclusivity rate (ensuring the method produces results for as many individuals as possible). Based on the results, Avent et al. [31] provided recommendations for interpreting and reporting classifications when using the original Walker [3] equations or the updated Garvin et al. [32] equations for sex estimation using traits of the skull. Unfortunately, both the Walker [3] and Garvin et al. [32] equations used ME scores, and, as discussed previously, recent studies have demonstrated unacceptably high rates of observer error for ME. However, their evidence‐based approach of establishing PPITs provides a roadmap for future improvements in sex estimation methods and is the first step toward best practice recommendations and standardization in the field.

### 1.3 Research goals

The primary goals of the current study were to (1) compare accuracy rates when using only the three innominate traits, only the four cranial traits, and the combined set of seven innominate and cranial traits using the RFM in MorphoPASSE and (2) use the approach of Avent et al. [31] to test for differences in accuracy rates when imposing PPITs to provide best practice recommendations for using the MorphoPASSE program. Secondarily, this study examined intra‐ and interobserver error rates and the relationship between cranial and innominate trait scores.

## 2 MATERIALS AND METHODS

### 2.1 Sample demographics and data collection

Data were collected from individuals curated in the Texas State University Donated Skeletal Collection (TXST, *n* = 87), Documented Skeletal Collection at the Maxwell Museum of Anthropology at the University of New Mexico (Maxwell, *n* = 58), University of Tennessee – Knoxville Donated Skeletal Collection (UTK, *n* = 81), and the John A. Williams Documented Human Skeletal Collection at Western Carolina University (WCU, *n* = 59). While forensic anthropology as a field is moving toward the estimation of population affinity rather than ancestry in casework, demographics for the sample used in this study are reported according to the ancestry or social race identities noted in the records kept by each institution (Table 1). The contemporary reference sample in MorphoPASSE does include individuals from both TXST and UTK; however, there is no overlap with the individuals selected for this study from those collections.

**TABLE 1:** Sample demographics.

	Asian	Black	Hispanic	Multiracial^a^	Native American or Alaskan native	White	Total
Female	0	10	9	0	1	114	134
Male	2	20	18	4	2	105	151
Total	2	30	27	4	3	219	285

*Note*: Ancestry groups are based on the demographic categories used in the documentation of each skeletal collection.

^a^ “Multiracial” includes individuals for whom records included more than one of the other ancestry categories included in this table.

Two observers independently collected innominate and cranial trait data for each individual in the sample while blinded to demographic information. The three innominate and four cranial traits were scored on an ordinal scale from 1 to 5 in accordance with the descriptions, figures, and recommendations included in the MorphoPASSE manual [13]. The left innominate was used except when unavailable or damaged, in which case the right innominate was substituted. Bilateral cranial traits (SOM and MP) were scored only on the left side, unless damaged, in which case the right side was substituted. MorphoPASSE [12] cautions against the use of ME scores due to high interobserver error. Therefore, only the four cranial traits (G, NC, MP, SOM) were used for this study. Individuals were selected for this study only if the observers recorded data for each of the three innominate traits and each of the four cranial traits.

Efforts were made during data collection to include individuals from as many ancestry groups as possible from each skeletal collection; however, most of the sample was White. Kruskal–Wallis tests were used to determine whether ancestry had any significant effects on trait score distributions. Due to low sample sizes, Asian, Native American or Alaskan Native, and Multiracial individuals were grouped together for this analysis. Tests were conducted on separated male and female subsamples.

### 2.2 Observer error

Both observers are experienced in collecting MorphoPASSE trait data; however, Observer 1 is the method creator. Observer 1's data could therefore be said to represent the best possible outcome when using MorphoPASSE, while Observer 2 represents a more typical experienced observer. To best reflect the use of MorphoPASSE in modern forensic casework, Observer 1's data were only used for the interobserver error study, while Observer 2's data were used for the intra‐ and interobserver error study and to compare accuracy rates and PPITs. Since this study included only individuals for which both observers recorded data for all seven traits, the interobserver error study utilized the full sample of 285 individuals. Intraobserver error for Observer 2 was tested on a random subsample of 50 individuals from the UTK collection (23F; 27M). Two trials of data collection were conducted ca. 1 week apart.

Observer error rates were calculated using squared‐weighted Cohen's Kappa, with strength of agreement based on Landis & Koch [33]. A weighted Kappa (wK) of <0 is poor agreement, 0.01–0.20 is slight agreement, 0.21–0.40 is fair agreement, 0.41–0.60 is moderate agreement, 0.61–0.80 is substantial agreement, and 0.81–1.00 is almost perfect agreement.

### 2.3 Trait correlations

Relationships between trait scores were examined using polychoric correlations. Polychoric correlations test the association between two ordinal variables, which are assumed to represent continuous latent variables. While each MorphoPASSE trait is scored on an ordinal scale of 1–5, the morphological variation within each trait is assumed to occur along a continuous spectrum. Pairwise correlation testing was conducted for all traits. Additionally, the innominate traits and the cranial traits were each summed to create new proxy variables to represent the overall relative robusticity of the innominate and cranium. The potential relationship between the innominate and cranial robusticity proxy variables was also tested with a polychoric correlation.

### 2.4 Accuracy rates and PP intervals

Sex was estimated in the MorphoPASSE GUI [12] using the RFM, with the following parameters selected to mimic US casework: contemporary temporal period, US region, and unknown ancestry. For each individual, sex was estimated through three model runs: using only the three innominate traits, using only the four cranial traits, and using the full set of all seven traits. Accuracy of the MorphoPASSE RFM using these three different trait combinations is as follows, along with the reference sample size and Kappa value: 92.0% for the innominate traits (*n* = 567; *K* = 0.8406), 88.9% for the cranial traits (*n* = 473; *K* = 0.7778), and 92.0% for combined innominate and cranial traits (*n* = 447; *K* = 0.84).

As noted above, MorphoPASSE does not currently provide a recommended PP threshold for reporting sex estimation, or not reporting it, for an individual. Therefore, five mutually exclusive PP intervals were examined (0.50–0.649, 0.65–0.749, 0.75–0.849, 0.85–0.949, and 0.95–1.00), and accuracy rates were calculated for each separate PP interval. For clarity, PP intervals will be subsequently referenced by their lower boundary (e.g., 0.50, 0.65, 0.75, 0.85, and 0.95). Accuracy rates were calculated as the proportion of individuals for whom sex was correctly predicted by MorphoPASSE within the PP interval. Binomial tests were used to examine whether the accuracy rate (proportion of correct predictions) of each PP interval was significantly different from random chance. The PP intervals that did have accuracy rates that were significantly different from random chance were further explored. Fisher's exact tests were conducted to test whether the PP intervals that did differ from random chance also differed significantly from each other. Since five binomial tests were performed for both the male and female subsamples, a Bonferroni correction of *p* ≤ 0.005 was implemented and applied for all PP interval and PPIT analyses.

### 2.5 PPITs

The accuracy results for the PP intervals were then used to develop several PPITs, which were created by condensing one or more of the adjacent PP intervals. For example, if the PP interval analyses showed no difference in accuracy rates between the 0.85 and 0.95 intervals, these intervals would be condensed into a single 0.85–1.00 PPIT. Accuracy rates for the newly created PPITs were then compared with a ‘no‐PPIT approach,’ as defined by Avent et al. [31], in which sex is predicted based on whether PPM or PPF was greater (i.e., whichever was >0.50). This no‐PPIT approach corresponds with MorphoPASSE, which currently does not provide a recommended PP threshold for classifying an individual. Applying a PPIT reduces the number of individuals from a given sample who can be classified; therefore, inclusivity rates were calculated as the proportion of individuals whose PP exceeded the applied PPIT and were therefore classified as either male or female, regardless of whether the classification was correct or incorrect. Fisher's exact tests were run to determine whether the PPITs differed significantly from each other or the no‐PPIT approach for accuracy rates or inclusivity. All statistical analyses were carried out using R and R Studio.

## 3 RESULTS

Distributions of innominate and cranial trait scores were tested for significant differences across ancestry groups in the separated male and female samples. Only G scores for the male sample were moderately different (*p* = 0.020). Visual analysis of boxplots showed that White males had a slightly higher mean G score compared with the Black, Hispanic, and grouped Asian, Native American or Alaskan Native, and Multiracial male samples. There were no significant differences in the score distributions for any other trait in the male sample, and there were no significant differences for any trait in the female sample (all *p* > 0.050). All ancestry groups were grouped into overall male and female samples for all remaining analyses.

### 3.1 Observer error

Interobserver error analyses show high levels of agreement between Observer 2 and the MorphoPASSE method creator (Observer 1). Agreement was almost perfect for NC (wK = 0.863), G (wK = 0.844), and VA (wK = 0.838), and was substantial for SPC (wK = 0.779), MP (wK = 0.743), SOM (wK = 0.667), and MA (wK = 0.614).

For the intraobserver error analyses, agreement was almost perfect for NC (wK = 0.874), G (wK = 0.913), MP (wK = 0.855), SOM (wK = 0.846), VA (wK = 0.84), and SPC (wK = 0.886), while agreement for the MA was moderate (wK = 0.531). All remaining analyses use only Observer 2's data.

### 3.2 Trait correlations

Initial polychoric correlation testing on the total sample found several significant pairwise correlations; however, visual analyses of scatterplots indicated that the significance of these results was likely due to the clustering of the male and female subsamples. Therefore, all correlations were re‐run on divided male and female samples.

Within the sex‐specific analyses, there were no significant pairwise correlations for either the male or female samples within the innominate traits or the cranial traits. The only moderately significant pairwise correlation was between VA and SOM in the female sample, which showed a slight negative correlation (*r*_*p*_ = −0.126, *p* = 0.027) (Table 2).

**TABLE 2:** Polychoric correlation values (*r*_*p*_ ) for pairwise trait comparisons.

	MA	VA	SPC	G	NC	SOM	MP
MA		−0.079	0.176	0.065	−0.026	−0.268	−0.170
VA	0.028		0.301	−0.008	−0.040	−0.033	0.040
SPC	0.120	0.208		0.0463	0.0406	0.0187	−0.068
G	−0.023	0.149	−0.030		0.193	0.233	0.132
NC	0.043	0.248	−0.148	0.160		0.0713	0.010
SOM	0.031	−0.126*	0.032	0.265	−0.132		0.125
MP	−0.082	−0.031	0.027	0.042	0.091	0.002	

*Note*: Values for the male sample are in shaded cells above the diagonal, and values for the female sample are in white cells below the diagonal.

* *p* < 0.050.

**FIGURE 1:**
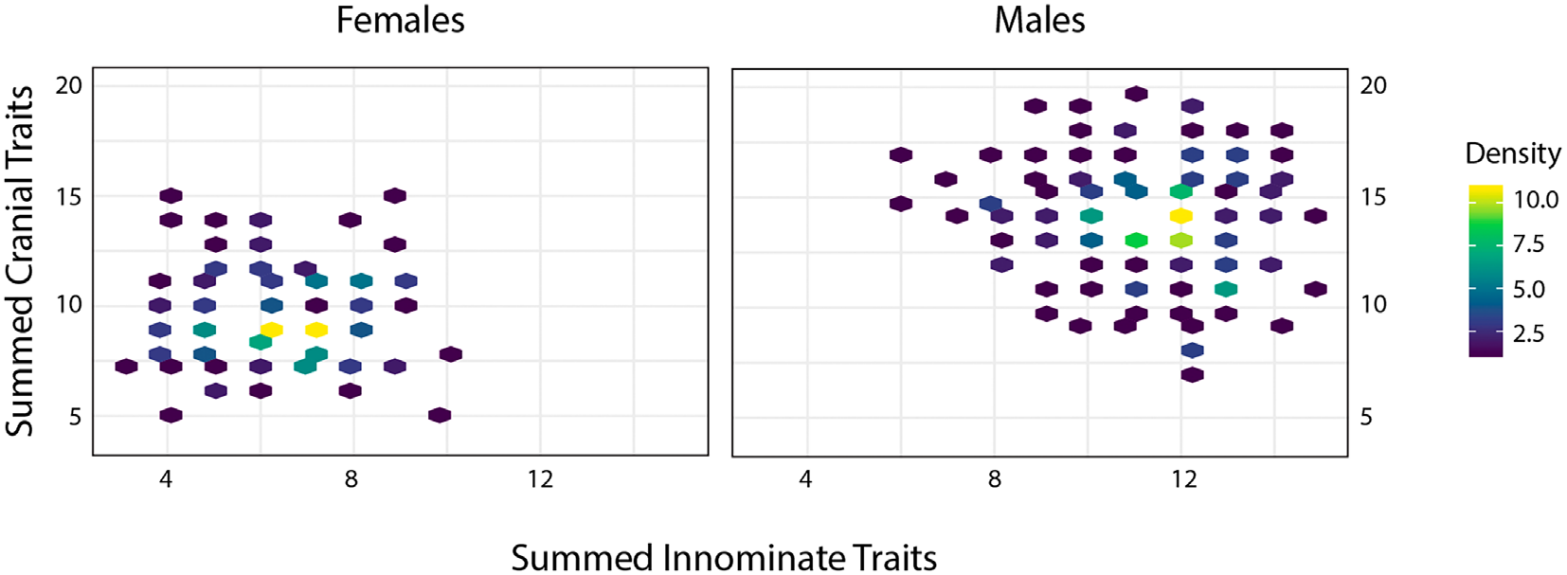
Hexbin plots comparing the summed total of the three innominate trait scores (*x*‐axis) with the summed total of the four cranial trait scores (*y*‐axis). The color of each hexagon indicates the density of individuals with a specific combination of summed scores, with yellow indicating the highest density of individuals.

Innominate trait scores and cranial trait scores were summed to create proxy variables to represent the overall relative robusticity of the innominate and cranium. The range, mean, and standard deviation for these robusticity proxy variables are provided in Table 3. The female sample had a smaller range, mean, and standard deviation for both the cranial and innominate robusticity proxy variables compared with the male sample. Neither the male (*r*_*p*_ = −0.0834, *p* = 0.934) nor female (*r*_*p*_ = −0.0160, *p* = 0.162) sample showed significant correlations between the proxy innominate and cranial variables. This suggests that an individual with a relatively overall gracile presentation of the innominate traits (i.e., a lower summed overall proxy score) would not necessarily have a similarly gracile presentation of the cranium, or vice versa. Similarly, an individual with a relatively overall robust appearance of the innominate traits (i.e., a higher summed proxy score) would not necessarily have a similarly robust appearance of the cranium, or vice versa. Figure 1 shows a broad distribution of summed innominate and cranial trait scores for both females and males.

**TABLE 3:** Range, mean, and standard deviation for the summed cranial and innominate robusticity proxy variables for males and females.

	Range	Mean (SD)
Cranial robusticity proxy		
Males	7–20	14.0 (2.51)
Females	5–15	9.43 (2.05)
Innominate robusticity proxy		
Males	6–15	11.3 (1.81)
Females	3–10	6.25 (1.54)

### 3.3 Accuracy rates and PP intervals

**FIGURE 2:**
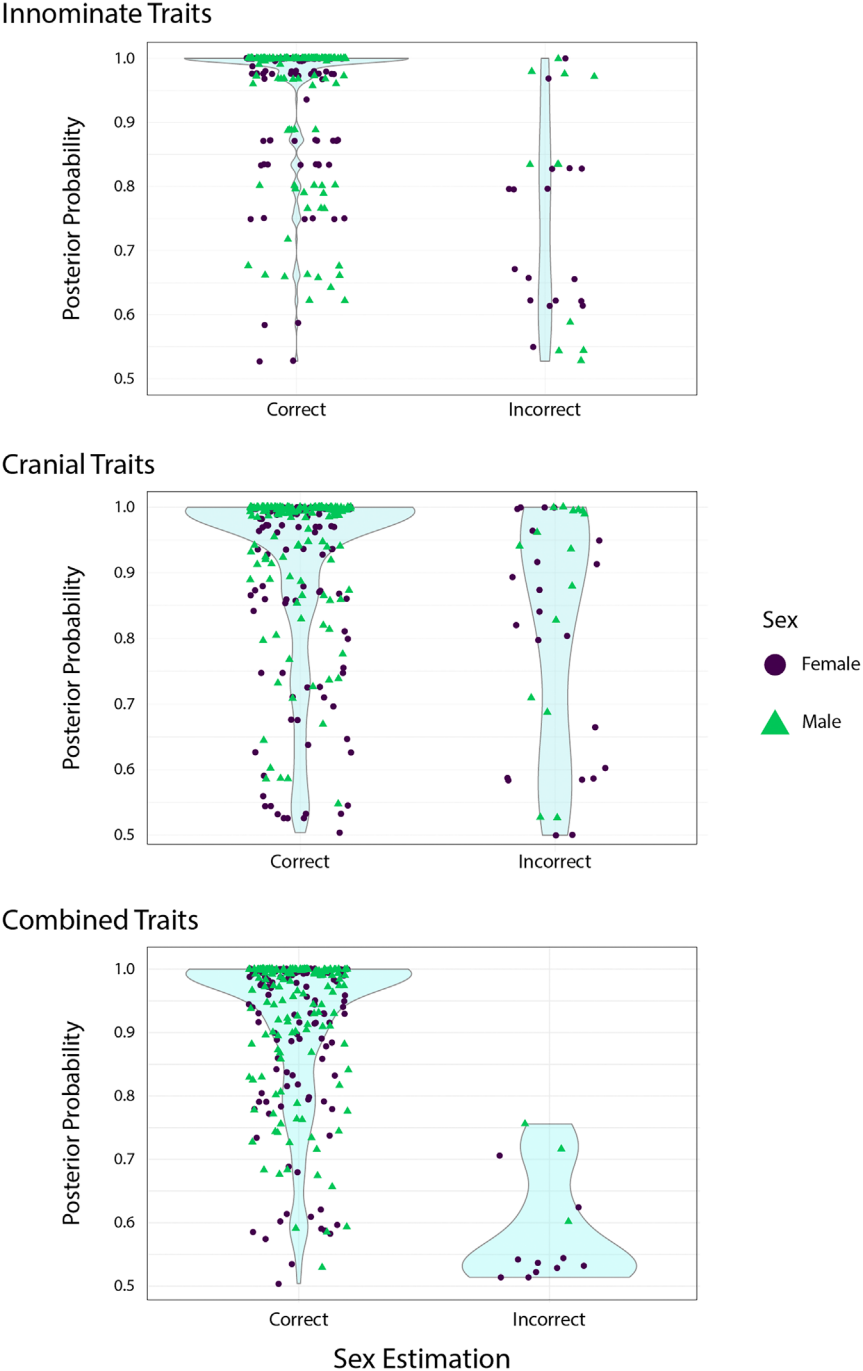
Violin density plots showing the distribution of PPs for correctly and incorrectly classified individuals for the innominate‐only model (top), cranium‐only model (middle), and combined model (bottom). Purple circles indicate females, and green triangles indicate males.

Figure 2 shows the distribution of PPs for correctly and incorrectly classified individuals using the innominate traits, cranial traits, and the combined set of traits, based on the current no‐PPIT approach of MorphoPASSE (i.e., using a threshold of >0.50 for classification). Notably, for both the innominate‐only and cranial‐only MorphoPASSE analyses, individuals were misclassified with PPs ranging from 0.501 to 1.0, demonstrating that misclassifications can occur even when the PP indicates a high degree of confidence in the classification. The results for two individuals produced both a PPM and a PPF of exactly 0.50 using the cranial traits, which were considered incorrect classifications. When using the MorphoPASSE model with the combined set of traits, incorrectly classified individuals exhibited a decreased range of PPs, from 0.501 to 0.75, demonstrating that all misclassifications had relatively low levels of confidence.

**FIGURE 3:**
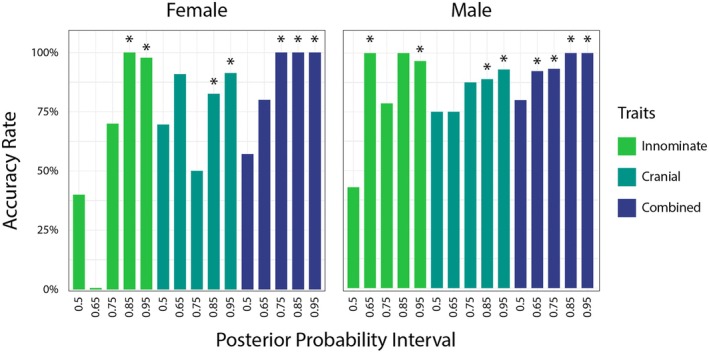
Bar graph comparing accuracy rates across PP intervals for females (left) and males (right). Each mutually exclusive interval is labeled by its lower boundary, with the 0.50 bar showing the accuracy rate for the 0.50–0.649 interval, the 0.65 bar showing the accuracy rate for the 0.65–0.749 interval, etc. Green bars show results when using the innominate traits, teal bars show results when using the cranial traits, and dark blue bars show results using the combined set of all seven traits. * indicates PP intervals for which the accuracy rate was significantly different from random chance (*p* < 0.005).

The distribution of PPs was also examined in terms of accuracy rates across the five mutually exclusive PP intervals tested (Figure 3; Table 4). The relationship between accuracy rate and PP interval is discussed separately for each of the three MorphoPASSE models.

**TABLE 4:** Distribution of sample within each mutually exclusive PP interval for each set of traits.

PP interval	Innominate‐only		Cranial‐only		Combined traits	
	Females	Males	Females	Males	Females	Males
0.50–0.649	10	7	23	8	21	5
0.65–0.749	3	8	11	8	5	13
0.75–0.849	20	14	8	8	16	15
0.85–0.949	11	5	23	27	25	27
0.95–1.00	90	117	69	100	67	91

#### 3.3.1 Innominate traits

Accuracy rates for both males and females in the 0.50, 0.65, and 0.75 PP intervals were not significantly different from chance. While the 0.65 PP interval for males did reach 100% accuracy, there were only eight known males falling within this interval, and the binomial test did not exceed the Bonferroni correction (*p* = 0.00781). Accuracy rates for the 0.85 and 0.95 intervals exceeded 96% for both females and males, although the accuracy rate for males at the 0.85 interval did not differ from chance, likely due to the low number of individuals within this interval (*n* = 5, *p* = 0.0625). There was no significant difference in accuracy rates between the 0.85 and 0.95 intervals for either males (*p* = 1) or females (*p* = 1), suggesting that these intervals provide similar levels of accuracy when estimating sex using only the innominate traits.

#### 3.3.2 Cranial traits

Accuracy rates for the 0.50, 0.65, and 0.75 intervals did not differ significantly from random chance for either males or females, while the accuracy rates for the 0.85 and 0.95 intervals did differ from chance. Like the innominate traits, there was no significant difference in accuracy rates between the 0.85 and 0.95 intervals for either males (*p* = 0.442) or females (*p* = 0.261). However, accuracy was lower, between 82.6%–91.3% for females and 88.9%–93.0% for males, in these intervals using only the cranial traits compared with the accuracy achieved with the innominate traits (>96%).

#### 3.3.3 Combined innominate and cranial traits

Only the 0.5 and 0.65 intervals for females and the 0.50 interval for males failed to differ significantly in accuracy from random chance when using the combined set of innominate and cranial traits. There was no difference in accuracy between the 0.75, 0.85, and 0.95 intervals for females or between the 0.65, 0.75, 0.85, and 0.95 intervals for males. These results suggest that similar levels of accuracy in sex estimation are possible at lower PP intervals when using the combined set of traits. Additionally, 100% accuracy was achieved for females in each of the 0.75, 0.85, and 0.95 intervals and for males in the 0.85 and 0.95 intervals. These results suggest that higher levels of accuracy are possible when using the combined set of traits.

### 3.4 PPITs

Based on analyses of accuracy rates within each PP interval as reported above, intervals were condensed into PPITs to further examine the effect of imposing a threshold on both accuracy and inclusivity of the overall sample in the final classifications. PPITs of 0.85–1.00 and 0.95–1.00 were tested for the innominate‐only and cranial‐only models, and PPITs of 0.75–1.00, 0.85–1.00, and 0.95–1.00 were tested for the combined model. These results were compared with accuracy rates with a no‐PPIT approach (0.501–1.00), when sex is predicted based on whether the PPM or PPF exceeded 0.50, regardless of magnitude. As before, for clarity in this section, PPITs will be referenced by their lower boundary (e.g., 0.75, 0.85, and 0.95).

#### 3.4.1 Innominate traits (0.85 and 0.95 PPITs)

Accuracy rates were high when using the no‐PPIT approach (87.3% females; 92.7% males) (Table 5), although higher accuracy rates were achieved with both tested PPITs. The accuracy rate for females with the 0.85–1.00 PPIT was significantly better than the no‐PPIT approach (98.0%; *p*_0.85_ = 0.00289). While accuracy was higher for the 0.95–1.00 PPIT (97.8%) compared with the no‐PPIT approach, the difference in accuracy rates was not significant with the Bonferroni correction (*p*_0.95_ = 0.00605), likely due to the lower rate of inclusivity. Interestingly, the accuracy rates for males when using the 0.85 and 0.95 PPITs (96.7% and 96.6%, respectively) were not significantly better than the no‐PPIT approach (*p*_0.85_ = 0.186; *p*_0.95_ = 0.193). This result was likely driven by the large number of individuals with PPs >0.85. However, the overall accuracy rates for the combined sample for the 0.85 and 0.95 PPITs (97.3% and 97.1%, respectively) were significantly better than the no‐PPIT approach (*p*_0.85_ = 0.00115; *p*_0.95_ = 0.00339). These results suggest that using a PPIT for the innominate model significantly improves overall accuracy rates. There were no significant differences between the accuracy rates for the 0.85 and 0.95 PPITs for either the divided or pooled sex sample (all *p* = 1.0), suggesting that the higher PPIT does not provide additional improvements in accuracy.

**TABLE 5:** Accuracy and inclusivity rates for PPITs using only the innominate traits.

Innominate traits						
PPIT	Female		Male		Overall	
	Accuracy	Inclusivity	Accuracy	Inclusivity	Accuracy	Inclusivity
No PPIT	87.3% (117/134)	100% (134/134)	92.7% (140/151)	100% (151/151)	90.2% (257/285)	100% (285/285)
0.85–1.00	98.0%* (99/101)	75.4%* (101/134)	96.7% (118/122)	80.8%* (122/151)	97.3%* (217/223)	78.2%* (223/285)
0.95–1.00	97.8% (88/90)	67.2%* (90/134)	96.6% (113/117)	77.5%* (117/151)	97.1%* (201/207)	72.6%* (207/285)

* Indicates accuracy or inclusivity rates that were significantly different from the no‐PPIT approach (*p* < 0.005).

Using a PPIT necessarily reduces inclusivity, or the proportion of individuals for whom sex can be predicted. The proportion of the sample that was able to be predicted was significantly lower for both the 0.85 and 0.95 PPITs for the divided and pooled sex samples compared with the no‐PPIT approach (all *p* < 0.005); however, there was no significant difference between the 0.85 and 0.95 PPITs, indicating that imposing the higher 0.95 PPIT does not significantly reduce the proportion of the sample for whom sex can be predicted beyond the 0.85 PPIT.

#### 3.4.2 Cranial traits (0.85 and 0.95 PPITs)

Accuracy rates were high when using the no‐PPIT approach (86.3% females; 90.1% males) (Table 6). While higher accuracy rates were achieved with both tested PPITs, these differences were not significant for any of the divided or pooled sex samples when compared with the no‐PPIT approach (all *p* > 0.005). This suggests that using a PPIT for the cranial model does not significantly improve accuracy, although this result was likely driven by the high number of individuals with PPs >0.85. There was also no significant difference in accuracy rate between the 0.85 and 0.95 PPITs, suggesting that the higher PPIT does not result in improved accuracy. Using a PPIT does, however, significantly reduce the proportion of the sample for whom sex can be predicted (all *p* < 0.005).

**TABLE 6:** Accuracy and inclusivity rates for PPITs using only the cranial traits.

Cranial traits						
	Female		Male		Overall	
	Accuracy	Inclusivity	Accuracy	Inclusivity	Accuracy	Inclusivity
No PPIT	86.3% (112/134)	98.5% (132/134)	90.1% (136/151)	100% (151/151)	87.0% (248/285)	99.3% (283/285)
0.85–1.00	89.1% (82/92)	68.6%* (92/134)	92.1% (117/127)	84.1%* (127/151)	90.9% (199/219)	76.8%* (219/285)
0.95–1.00	91.3% (63/69)	51.1%* (69/134)	93.0% (93/100)	66.2%* (100/151)	92.3% (156/169)	59.3%* (169/285)

* Indicates accuracy or inclusivity rates that were significantly different from the no‐PPIT approach (*p* < 0.005).

#### 3.4.3 Combined innominate and cranial traits (0.75, 0.85, and 0.95 PPITs)

Accuracy rates when using the combined set of innominate and cranial traits were higher than the innominate‐only and the cranial‐only models for all the examined PPITs (Table 7). For females, accuracy rates for the 0.75 (100%; *p* = 0.00257) and 0.85 (100%; *p* = 0.000624) PPITs were significantly higher compared with the no‐PPIT approach. While the female accuracy rate for the 0.95 PPIT was not significantly different from the no‐PPIT approach based on the Bonferroni correction (100%; *p* = 0.0326), this was likely due to the high accuracy of the no‐PPIT approach (92.5%) combined with the smaller number of individuals exceeding a PP of 0.95. None of the male accuracy rates for the 0.75 (99.2%), 0.85 (100%), or 0.95 (100%) PPITs were significantly different from the no‐PPIT approach (all *p* > 0.1), which was also likely due to the high accuracy of the no‐PPIT approach (98.0%). However, the overall accuracy rates for the pooled sample were significantly different from the no‐PPIT approach for the 0.75 (*p* = 0.00244) and 0.85 (*p* = 0.000907) PPITs. As with the female subsample, the overall accuracy rate for the 0.95 PPIT failed to reach significance (*p* = 0.00552), likely due to the high accuracy of the no‐PPIT approach and lower inclusivity of the 0.95 PPIT. These results suggest that in the total sample, using a PPIT for the combined trait model can significantly improve accuracy rates. Sex bias was also reduced from 5.5% with the no‐PPIT approach to 0.8% with the 0.75 PPIT and 0% with the 0.85 and 0.95 PPITs. There were no significant differences between the accuracy rates for the 0.75, 0.85, and 0.95 PPITs for either the divided or pooled sex samples (all *p* = 1.0), suggesting that imposing a PPIT higher than 0.75 does not provide additional improvements in accuracy.

**TABLE 7:** Accuracy and inclusivity rates for PPITs using the combined innominate and cranial traits.

Combined innominate and cranial traits						
	Female		Male		Overall	
	Accuracy	Inclusivity	Accuracy	Inclusivity	Accuracy	Inclusivity
No PPIT	92.5% (124/134)	100% (134/134)	98.0% (148/151)	100% (151/151)	95.4% (272/285)	100% (285/285)
0.75–1.00	100%* (108/108)	80.6%* (108/134)	99.2% (132/133)	88.1%* (133/151)	99.6%* (240/241)	84.6%* (241/285)
0.85–1.00	100%* (92/92)	68.7%* (92/134)	100% (118/118)	78.1%* (118/151)	100%* (210/210)	73.7%* (210/285)
0.95–1.00	100% (67/67)	50%* (67/134)	100% (91/91)	60.3%* (91/151)	100% (158/158)	55.4%* (158/285)

* Indicates accuracy or inclusivity rates that were significantly different from the no‐PPIT approach (*p* < 0.005).

The proportion of the sample that was able to be predicted using a PPIT was significantly lower for all tested PPITs for the divided and pooled sex samples compared with the no‐PPIT approach (all *p* < 0.005). While the 0.75 PPIT was not significantly different from the 0.85 PPIT for the divided male (*p* = 0.0308) and female (*p* = 0.0348) samples, all other comparisons for the divided and pooled samples resulted in significant reductions in inclusivity with increasing PPITs (all *p* < 0.005). This indicates that the higher PPITs do reduce the number of individuals for whom sex can be predicted.

## 4 DISCUSSION

MorphoPASSE presented a significant step forward in the progression of sex estimation methods by using a more advanced statistical algorithm, accommodating missing data, and allowing users to combine morphological data from the innominate and the cranium into a single prediction model. While this provided a way to streamline sex estimation, individual users are still required to decide whether to combine traits and how to interpret the MorphoPASSE output file. The ability to make these decisions is premised on an understanding of the individual traits and how they relate to each other. Information to support this decision‐making process has been sorely lacking; however, the results of this study can now provide insight for best practice recommendations with respect to using MorphoPASSE for sex estimation in modern forensic casework.

### 4.1 Observer error

There are few published reports of either intra‐ or interobserver error for the MorphoPASSE traits available for comparison. Multiple observer error studies have been conducted on both the Walker [3] cranial traits and the Klales et al. [11] innominate traits (see [34], Tables 1 and 2). However, as noted previously, the MorphoPASSE manual made significant revisions to both the Walker [3] and Klales et al. [11] trait descriptions, limiting the utility of comparing observer error between the methods. No other studies were found that measured observer error for the innominate traits when specifically using the revised MorphoPASSE descriptions. In this study, agreement for VA and SPC was almost perfect for both intra‐ and interobserver error. Agreement for MA was substantial in the interobserver error study and was moderate in the intraobserver error study.

Kotěrová et al. [20] was the only peer‐reviewed report of interobserver error rates for the four cranial traits with the revised MorphoPASSE descriptions; however, error rates were tested using a linear‐weighted Cohen's Kappa (lK). This study used squared weighting, as this approach penalizes small disagreements (e.g., a difference of 1 or 2 between scores) less than a linear wK but exponentially accelerates the penalty for larger disagreements (e.g., a difference of 3 between scores). Using the Landis and Koch [33] scale, the interobserver error study in Kotěrová et al. [20] showed substantial agreement for G (lK = 0.717) and NC (lK = 0.638) and moderate agreement for SOM (lK = 0.529) and MP (lK = 0.498). Results in this study using squared‐weighted Cohen's Kappa were significantly higher, with G and NC showing almost perfect agreement (wK = 0.844 and 0.863, respectively) and SOM and MP showing substantial agreement (wK = 0.667 and 0.743, respectively). However, without access to the raw data of Kotěrová et al. [20] it is impossible to determine the effect that the choice of weighting schemes had on the results. The intraobserver error results in this study showed almost perfect agreement for all four cranial traits.

### 4.2 Trait correlations

None of the cranial traits were significantly correlated with each other. While Garvin et al. [32] found some significant pairwise correlations between cranial traits, the correlation values were low, and there was no pattern in trait correlations among the studied populations. The difference in findings between these studies could be due to the use of different trait descriptions (MorphoPASSE [12] versus Walker [3]) or the use of different statistics (polychoric versus Spearman rank correlation). Garvin et al. [32] did note that it was not unusual for an individual to exhibit inconsistent scores for the cranial traits due to different selective and developmental pressures. While some cranial traits are muscular attachment sites, others might be subjected to unknown functional or structural pressures.

Similarly, none of the innominate traits were found to be significantly correlated with each other in this study. Klales et al. [11] had also performed polychoric correlation testing on the innominate traits and found significant relationships between all three traits. However, these tests were conducted on the pooled sex sample; therefore, it is likely that the results captured a false correlation based on the clustering of males and females. The lack of significant correlation between VA and SPC found in this study, particularly in females, is somewhat surprising in light of the sexually dimorphic growth of the innominate. The development of a ridge of bone at the site of attachment for the hip adductors and the buildup of bone producing a squared off appearance of the pubis are both considerations of VA scoring and are both related to the elongation of the pubis in females during puberty [35, 36, 37, 38]. Similarly, the development of concavity along the inferior pubic ramus, scored as SPC, is also related to pubis elongation [35]. Since the relative thickness of the ischiopubic ramus used in MA scoring is, at least in part, related to the attachment of the erectile bodies of the penis and clitoris [35, 39] rather than elongation of the pubis, the lack of correlation between MA and either VA or SPC score is less surprising.

This study additionally created proxy variables to capture overall cranial and innominate robusticity to examine intraindividual variation. Relative robusticity of these skeletal elements has been quantified in previous research through geometric morphometrics. For example, Best et al. [19] compared relative degrees of robusticity in the cranium and innominate using coordinate landmark data, finding a lack of significant intraindividual correlations between either the shape or degree of sexual expression in the innominate and cranium. Similarly, in this study, only VA and SOM showed a slight negative correlation in females, and there was no significant correlation between the summed proxy variables for innominate and cranial relative robusticity. These results make sense considering the variable growth and developmental rates of the innominate and cranium and the differential selective pressures acting on these structures [35, 40]. Developmental timing influences the degree of sexual dimorphism, with later growing structures, like the innominate, exhibiting more sexual dimorphism than earlier growing structures, like the cranium and facial region, due to different functional requirements (e.g., locomotion/reproduction vs. neural/mastication respectively) [41].

Overall, the lack of significant intraindividual correlation between the innominate and cranium found in this study supports the conclusions of Best et al. [19]. There is intraindividual variation in terms of sexual expression between the cranium and innominate. In general, females do have lower cranial and innominate trait scores than males; however, within each sex, a more robust cranium cannot be assumed to be associated with a more robust innominate. Therefore, in assemblages of commingled remains, relative degrees of robusticity may be useful for initially sorting individual elements, but reassociating a cranium and innominate should be based on more than comparable levels of trait scores. Further, these results support using both the cranium and innominate for morphological sex estimation when both elements are present.

### 4.3 Accuracy rates and PPITs

The results of this study demonstrate that combining the innominate and cranial traits with the MorphoPASSE RFM produces higher accuracy rates than either skeletal region alone. Even when using a no‐PPIT approach, the combined trait model produced the highest overall accuracy rate (95.4%), compared with the cranial‐only (87.0%) and innominate‐only (90.2%) models. However, perhaps the more interesting finding was that using the combined set of traits reduced the chance of making an incorrect sex classification with high confidence (i.e., a high PP).

#### 4.3.1 Innominate‐only model

Even though the innominate is widely recognized as the most sexually dimorphic element, multiple individuals in this study would have been misclassified with a high degree of confidence when using only the three innominate traits. Using a no‐PPIT approach, 28 individuals (nearly 10% of the total study sample) would have been misclassified using only the innominate. While a no‐PPIT approach does not conflict with the current MorphoPASSE manual, most practitioners would not provide a sex classification in a forensic report based on a method that produced a PP of 0.51. This study provides evidence that the 0.85 and 0.95 PP intervals produced sex classifications that were significantly different from chance and improved accuracy compared with a PP threshold of 0.50. Imposing a PPIT of 0.85 improved overall accuracy to 97.3%. In addition to improving accuracy, using a 0.85 PPIT also reduced sex bias, from 5.4% down to 1.3%. However, these improvements come at the cost of only being able to estimate sex for 78.2% of the sample, reducing the inclusivity of the method and still resulting in six individuals being misclassified with high levels of confidence (PP >0.95).

#### 4.3.2 Cranium‐only model

Using a no‐PPIT approach, the cranial trait model achieved an 87.0% accuracy rate, which was slightly lower than the innominate trait model (90.2%). Higher PP intervals were explored and found to be significantly different from chance in terms of accuracy. Yet, higher PPITs did not produce accuracy rates that were significantly different from the no‐PPIT approach, although this was likely driven by the high number of individuals with PPs greater than 0.95. Kotěrová et al. [20] similarly compared accuracy rates using the four cranial traits with several PPITs for classification; however, no statistical testing was conducted to determine if the selected thresholds provided any significant improvement. Of additional note, in the current study, even when applying a PPIT of 0.95, 13 individuals were misclassified.

#### 4.3.3 Combined trait model

In contrast, the combined trait model achieved near perfect accuracy with a PPIT of 0.75 – only a single known male was misclassified (PP = 0.756). Imposing a PPIT of 0.85 produced a 100% accuracy rate for both males and females, although this higher PPIT did result in a significantly lower number of individuals for whom sex would be predicted (210/285 individuals classified using the 0.85 PPIT vs. 241/285 individuals classified using the 0.75 PPIT). The combined trait model was the only model to reach a 100% accuracy rate.

With the PPIT approach tested in this research, the combined model was successful at correcting all misclassifications using either the innominate or cranium alone, either by producing a correct classification or leaving the individual unclassified for failure to meet the PPIT. Using the 0.85 PPIT with only the innominate traits, six individuals were incorrectly classified; when using the 0.75 PPIT combined model, all 6 of these individuals failed to meet the threshold and were not classified. Using the 0.85 PPIT with only the cranial traits, 20 individuals were incorrectly classified; when using the 0.75 PPIT combined model, 12 of these individuals were correctly classified, and the remaining eight individuals failed to meet the threshold and were not classified.

### 4.4 Standard 090: Sex estimation in forensic anthropology

Many factors influence the decision of which sex estimation method to select for any given case – the skeletal elements present for analysis, pathological or taphonomic damage to the remains, access to analytical equipment or proprietary software, and user experience and confidence in a given method are all part of the consideration [1]. The Standard for Sex Estimation in Forensic Anthropology (“Standard 090”) [27] states that, if multiple methods are available and applicable to the remains being examined, “the method(s) with the greatest accuracy shall be given greater consideration when evaluating the totality of the evidence.” However, as discussed, overall method accuracy must be balanced with considerations of PPs, yet Standard 090 is silent on an approach or recommendation for PP thresholds.

Standard 090 states that the degree of certainty for a classification should be expressed in a report either numerically or using qualitative qualifiers [27]. The examples of potential numerical qualifiers were correct classification rates and method accuracies, with no discussion of PPs. Examples of potential qualitative qualifiers include the arbitrary “probable,” with the statement that “sex estimates should be reported as female, male, probable female, probable male, or undetermined.” Again, Standard 090 is silent as to how an analyst is to determine the cutoff between undetermined and probable and between probable and male or female. This is further complicated, as Klales [16] notes, by the current lack of “generally accepted procedures for addressing bias, reliability, and validity of the methods available in forensic anthropology.” Even the term “undetermined” is questionable in light of current views on sex estimation. Standard 090 states that “estimation” is “largely interchangeable with determination” [27]; however, “determination” implies a near‐certain level of confidence that is impossible to obtain with current sex estimation methods [16]. Sex “estimation” better reflects the actual practice of forensic anthropology. Therefore, when a sex estimation is not supported by a method, the case report should state that the sex of the individual “could not be estimated” rather than reporting sex as “undetermined.”

Many historical sex estimation methods used majority rule approaches, linear regression analysis, or simple cutoff values, which lacked statistical estimates of confidence in the classification. Most modern methods do provide this statistical estimate, generally in the form of a PP; however, currently available methods stop short of providing explicit guidance on how to interpret and report method results based on the calculated PP and instead leave the interpretation up to the individual analyst or the stakeholders reading the report. Many forensic anthropology case reports also fail to include the appropriate information for stakeholders and forensic anthropology peer‐reviewers to accurately interpret the reported results. For appropriate interpretation, when providing a sex estimate based on MorphoPASSE results, the report should include which traits and parameters were utilized for the model (time period, region, ancestry/population affinity, and statistical model), the model accuracy/Kappa value, the reference sample size, and the PPIT, in addition to the sex classification and associated PP (as a percentage). These details should be included in the report itself and not just in the attached bench notes or supplemental files.

### 4.5 Limitations

While this study represents an important step toward standardizing the use of MorphoPASSE for sex estimation, several limitations must be recognized. First, this study only included individuals with data for the complete set of seven innominate and cranial traits, which is a “best case scenario” for forensic casework. The RFM selects the most informative input variables. Variable importance is based on the mean decrease in the Gini coefficient, with the most important variable in the model being the one with the highest mean decrease in the out‐of‐bag error estimate [13]. However, the relative order of variable importance will change depending on which combination of traits is being analyzed. For example, when analyzing only the three innominate traits, MA is the least important trait, yet when analyzing all seven traits, MA is more important in the RFM than MP, NC, and SOM. Therefore, additional studies could be completed to explore how accuracy and PPITs might be affected by using different combinations of traits. MorphoPASSE is notable for its ability to accommodate missing data, so exploration in this area is warranted.

Second, MA achieved only a moderate level of agreement in the intraobserver error study, despite reaching a substantial level of agreement in the interobserver error study. Previous observer error studies using MA based on Klales et al. [11] reported substantial or nearly perfect agreement (see [34], Table 2). Notably, the original Klales et al. [11] study reported moderate agreement for SPC, which was deemed acceptable. While many studies utilize the Landis & Koch [33] categorization scheme for levels of agreement using wK, forensic anthropology lacks guidelines on what is an acceptable level of agreement. Determining acceptable levels of agreement for morphological trait scoring will depend on the scale of trait scoring, the statistical method used to quantify observer error, the statistical model used by the estimation method, and how input variables are weighted. Future studies will explore the different statistics that have been used to quantify observer error for morphological traits scoring and the effect that different levels of agreement have on method accuracy.

Third, it must be emphasized that these results are based on selecting the specific MorphoPASSE model parameters used in this study: contemporary temporal period, US region, and unknown ancestry. Future studies could explore whether the PPIT recommendations are also supported for other parameter options, as practitioners may prefer to use MorphoPASSE without limiting the reference sample to the US region.

Finally, a critique of the Klales et al. [11] method is that many of the subsequent validation studies have either been performed or co‐authored by Dr. Klales, one of the method creators, or by individuals who have been trained by Dr. Klales, (e.g., [8, 42, 43]). As a result, there is a legitimate question of whether the results of these studies can provide a realistic reflection of the method's performance when trait scoring is completed by someone not trained directly by Dr. Klales. This study focused on the traits as defined and described in the MorphoPASSE manual [13], using data collected by Observer 2. While Observer 2 did receive training in scoring the traits as defined in Klales et al. [11], Walker [3], and the MorphoPASSE manual [13] from other individuals, further training and collaborative work related to MorphoPASSE have been conducted with Dr. Klales. Therefore, additional studies conducted by individuals who have not received any direct training from Dr. Klales are highly encouraged to further test both the trait definitions and accuracy of MorphoPASSE.

## 5 CONCLUSION

MorphoPASSE presented a significant step forward in the progression of sex estimation methods by allowing users to combine morphological data from the innominate and the cranium into a single sex prediction model, with the user able to select the most appropriate parameters (region, ancestry, temporal period). However, the field of forensic anthropology has lacked evidence‐based guidance on how to translate the results of a sex estimation method and associated statistical levels of confidence in the classification into a forensic report. For both the innominate‐only and cranial‐only MorphoPASSE models, accuracy rates for the 0.85 and 0.95 PP intervals were significantly different from chance. Therefore, individuals whose classification results in a PP <0.849 should be reported as “could not be estimated.” While using a PPIT for the cranial‐only model did not result in statistically better accuracy rates than a no‐PPIT approach, PP intervals <0.85 did not produce accuracy rates better than chance, which ultimately supports the use of a PPIT. The results of this study support the use of a 0.85–1.00 PPIT for both the innominate‐only and cranial‐only models, and individuals exceeding this PPIT should be reported as consistent with the male or female MorphoPASSE reference samples.

The combined trait model showed overall higher accuracy rates and the ability to make accurate classifications with a lower PPIT. Accuracy rates differed significantly from chance for each of the 0.75, 0.85, and 0.95 PP intervals, and the accuracy rate for the 0.75, 0.85, and 0.95 PPITs was significantly better than the no‐PPIT approach. Since there was no significant difference in accuracy between the 0.75, 0.85, and 0.95 PPITs, the condensed 0.75 PPIT is recommended. Although using the 0.75 PPIT still resulted in one misclassification, this approach was able to classify a larger proportion of the sample than higher PPITs. If both the innominate and cranium are present and all traits can be scored, the combined trait model is recommended using a PPIT of 0.75. Individuals whose classification results in a PP less than 0.749 should be reported as “could not be estimated.” If the PP is greater than or equal to 0.75, the individual should be reported as consistent with the male and female reference samples. Notably, Avent et al. [31] provided recommendations on when the “probable” qualifier for a male or female classification was statistically supported using the Walker [3] or Garvin [32] sex estimation method. In the current study, since there were no PP intervals that were significantly different from chance and significantly different from higher PP intervals, there is no support, from a statistical standpoint, for using the “probable” qualifier for a MorphoPASSE sex estimation using any of the models tested in this research.

Use of these recommendations and PPITs should standardize both the use of MorphoPASSE and the reporting of sex estimation results based on MorphoPASSE in forensic reports. Reports should include a list of the traits used in analysis and all model parameters selected, notation of the model accuracy with the sample size and Kappa value included, and both the PP reported for the individual being analyzed and the applied PPIT.

## FUNDING INFORMATION

This research was funded by the National Science Foundation Grant No. 2214747 (co‐funded by the National Institute of Justice DOJ‐NIJ‐22‐RO‐0007).

## CONFLICT OF INTEREST STATEMENT

The authors have no conflicts of interest to report.

## DISCLAIMERS

Any opinions, findings, and conclusions or recommendations expressed in this material are those of the author(s) and do not necessarily reflect the views of the National Science Foundation, the Department of Justice, or the National Institute of Justice.

## Data Availability

The data that support the findings of this study are available from the corresponding author upon reasonable request.
